# A Theoretical Investigation of the Relationship between Structural Equation Modeling and Partial Correlation in Functional MRI Effective Connectivity

**DOI:** 10.1155/2009/369341

**Published:** 2009-08-25

**Authors:** Guillaume Marrelec, Habib Benali

**Affiliations:** ^1^Inserm, U678, Laboratoire d’Imagerie Fonctionnelle, F-75013 Paris, France; ^2^UPMC Univ Paris 06, UMR-S 678, Laboratoire d’Imagerie Fonctionnelle, F-75013 Paris, France

## Abstract

An important field of blood oxygen level dependent (BOLD) functional magnetic resonance imaging (fMRI) is the investigation of effective connectivity, that is, the actions that a given set of regions exert on one another. We recently proposed a data-driven method based on the partial correlation matrix that could provide some insight regarding the pattern of functional interaction between brain regions as represented by structural equation modeling (SEM). So far, the efficiency of this approach was mostly based on empirical evidence. In this paper, we provide theoretical fundaments explaining why and in what measure structural equation modeling and partial correlations are related. This gives better insight regarding what parts of SEM can be retrieved by partial correlation analysis and what remains inaccessible. We illustrate the different results with real data.

## 1. Introduction

Blood oxygen level dependent (BOLD) functional magnetic resonance imaging (fMRI) is an imaging technique that allows to dynamically and noninvasively follow metabolic and hemodynamic consequences of brain activity [1, 2]. Since Biswal et al. [3], an increasing number of studies have suggested that fMRI data could be used to explore how brain regions interact to perform functional tasks. A key concept in investigation of functional brain interactions is effective connectivity, which has been defined as the influence that regions exert on one another [4]. 

 Path analysis, or structural equation modeling (SEM), has been the major way to examine effective connectivity in fMRI [5–7]. Starting from a set of *D* regions, a model is set a priori that expresses the time course *z*_*i*_(*t*) of each region as a linear function of the time course of other regions

(1) $$z_{i}\left(t\right)=\underset{j\neq i}{\sum }\lambda_{ij}z_{j}\left(t\right)+e_{i}\left(t\right),$$

with some coefficients *λ*_*ij*_ being constrained to 0, the others are free to vary. *λ*_*ij*_ quantifies the strength that region *j* exerts on region *i*. Setting an SEM is equivalent to defining a directed graph, where each node stands for a region, a given arrow *j* → *i* is present if and only if the corresponding coefficient *λ*_*ij*_ is not constrained to zero, and, finally, *λ*_*ij*_ represents the intensity of arrow *j* → *i*. Once the structural model is completely set, the unconstrained coefficients *λ*_*ij*_ are estimated. To this aim, the model covariance matrix **Σ**, which is a function of the parameters, is compared to the sample covariance matrix **S** using a discrepancy function that is minimized [8, 9]. In fMRI data analysis, the following maximum likelihood function is often used [7]:

(2) $$l\left(\Sigma \right)=\text{tr}\left(S\Sigma^{-1}\right)-\ln\;\left|\Sigma^{-1}S\right|-D,$$

where tr(·) stands for the standard matrix trace function. The major flaw of this approach is that it requires the prior definition of a structural model, that is, of regions and arrows, each arrow requiring itself information regarding connection and direction. By contrast, information regarding the functional interactions present within the network of interest is likely to be scarce, since it is often the very reason why an fMRI study of effective connectivity is carried out. This is all the more problematic that the approach does not really provide any clear way to challenge the model or to provide information relative to where or how the model under investigation could be improved. 

 We recently proposed a novel approach to gain insight on effective connectivity. We first showed that, unlike marginal (i.e., regular) correlation, conditional correlation could account for many patterns of interaction as modeled by SEM [10, 11]. We then proposed to focus on a specific set of conditional correlations, namely partial correlations [12]. Given a set of *D* regions, denoted by ℝ, and a variable *y*_*i*_ associated to each region *i* (of which *z*_*i*_(*t*) mentioned in (1) is a realization), the method estimates the partial correlation of any region pair (*i*, *j*) given the set of *D* − 2 remaining regions,

(3) $$\text{Corr}\left[y_{i},y_{j}\;|\;y_{\mathbb{R}\setminus \{i,j\}}\right].$$

On both real [13] and synthetic data [14], it was observed that a large partial correlation value between two regions was often associated with the presence of an effective connectivity between these regions. However, the reason for such a behavior remained unclear. In the present paper, we further delve into the relationship between SEM and partial correlation in order to better understand why and in what measure partial correlation can extract information that is relevant for effective connectivity analysis. To this aim, we provide a theoretical relationship between SEM and partial correlation through the computation of the inverse covariance matrix (also-called concentration or precision matrix). To illustrate the results so obtained, we use a dataset on which SEM analysis has already been performed and published [7].

## 2. From SEM to Partial Correlation

### 2.1. Bullmore et al. [7] SEM Study

We here quickly recall the essentials of a previous study on which our investigation of partial correlation relies. For more detail, we refer the reader to Bullmore et al. [7]. The study focused on *D* = 5 left hemispheric cortical regions of interest: the ventral extrastriate cortex (VEC), the prefrontal cortex (PFC), the supplementary motor area (SMA), the inferior frontal gyrus (IFG), and the inferior parietal lobule (IPL). Each region was associated to a time course for a total of five time courses of length *T* = 96 time samples. The sample marginal and partial correlation matrices corresponding to these time courses are reported in Table 1. The time courses were a group average over the subjects, and the correlation matrix corresponds to the correlations of the averaged time series.

**Figure 1:**
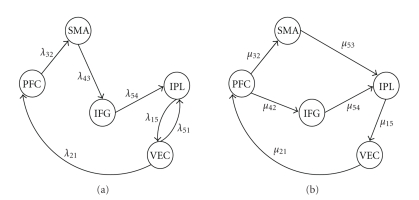
Structural models and path coefficients corresponding to the theoretically preferred (a) and best fit (b) models (from [7]).

 A plausible structural model, henceforth referred to as the “theoretically preferred model” (or “TP”), was proposed and is represented in Figure 1(a). Using the correlation matrix of Table 1, a procedure implemented in the LISREL proprietary software package (http://www.ssicentral.com/lisrel/) computed a so-called “best fit” model from the data, henceforth referred to as such (or “BF”) and represented in Figure 1(b). While similar in some ways, the two models had different features:

- VEC → IPL and SMA → IFG were present in the theoretically preferred model but were not selected in the best fit model;
- PFC → IFG and SMA → IPL were absent in the theoretically preferred model but appeared in the best fit model.

We now go back to a different perspective. Indeed, the structure of any SEM entails specific constraints on the covariance matrix, as well as other matrices characteristic of the process, such as the concentration matrix and the marginal and partial correlation matrices.

### 2.2. SEM Modeling

Generally speaking, a structural model can be defined in matrix form as

(4) y=Ky+e,

where **y** is the *D*-dimensional variable characterizing the state of each region and **e** is a temporally independent and identically distributed (i.i.d.) Gaussian noise with diagonal covariance matrix. **K** = (*K*_*ij*_)_*i*,*j*=1,…,*D*_ contains the path coefficients. The *N* time samples (**z**(*t*_*n*_))_*n*=1,…,*N*_, where **z**(*t*_*n*_) is the signal measured in each of the *D* regions at time *t*_*n*_, are supposed to be *N* i.i.d. realizations of **y**. The matrices corresponding to the theoretically preferred and the best fit models are, respectively, given by (see also Figure 1)

(5) $$\begin{array}{ll} K_{\text{TP}} & =\left(\begin{array}{cccccc} 0 & 0 & 0 & 0 & 0 & \lambda_{15}\\ \lambda_{21} & 0 & 0 & 0 & 0 & 0\\ 0 & \lambda_{32} & 0 & 0 & 0 & 0\\ 0 & 0 & \lambda_{43} & 0 & 0 & 0\\ \lambda_{51} & 0 & 0 & \lambda_{54} & 0 & 0 \end{array}\right),\\ K_{\text{BF}} & =\left(\begin{array}{cccccc} 0 & 0 & 0 & 0 & 0 & \mu_{15}\\ \mu_{21} & 0 & 0 & 0 & 0 & 0\\ 0 & \mu_{32} & 0 & 0 & 0 & 0\\ 0 & \mu_{42} & 0 & 0 & 0 & 0\\ 0 & 0 & \mu_{53} & \mu_{54} & 0 & 0 \end{array}\right). \end{array}$$

### 2.3. SEM and Covariance

Classically, we further assume that the noise **e** of (4) is composed of spatially and temporally independent Gaussian variables with diagonal covariance matrix:

(6) $$\text{Var}\left[e\right]=V=\left(\begin{array}{ccc} V_{1} & & 0\\ & \ddots & \\ 0 & & V_{D} \end{array}\right).$$

Since (4) rereads **y** = (**I** − **K**)^−1^ **e**, where **I** stands for the *D*-dimensional unit matrix, it is straightforward to show that **y** is also Gaussian distributed with covariance matrix [15]

(7) $$\Sigma ={\left(I-K\right)}^{-1}V{\left[{\left(I-K\right)}^{-1}\right]}^{\text{t}},$$

where “t” stands for matrix transposition. Since **K** is a function of the path coefficients, so is **Σ** This relationship is central to SEM analysis, for most methods rely on comparing the covariance matrix **Σ** implied by a structural model to the data sample covariance matrix using normal theory maximum likelihood—leading to the discrepancy function of (2)—, generalized least squares, or ordinary least squares [8, 9]. Note that, in (2), **Σ** only appears through its inverse **Υ** = Σ^−1^. **Υ** is called the concentration, or precision, matrix and it is on this matrix that we will focus to get a better understanding of the data structure.

### 2.4. SEM and Concentration

Indeed, **Υ** has intriguing structural properties when related to a structural model. Using (7), this matrix is given by

(8) $$\Upsilon ={\left(I-K\right)}^{\text{t}}V^{-1}\left(I-K\right).$$

**V** being a diagonal matrix, the expression for each element *Υ*_*ij*_ of the concentration matrix can easily be expanded as

(9) $$\Upsilon_{ij}=\underset{l}{\sum }\frac{\left(\delta_{li}-K_{li}\right)\left(\delta_{lj}-K_{lj}\right)}{V_{l}}.$$

Given that *K*_*ii*_ = 0, the previous equation yields

(10) $$\Upsilon_{ii}=\frac{1}{V_{i}}+\underset{l\neq i}{\sum }\frac{K_{li}^{2}}{V_{l}},$$

and, for *i* ≠ *j*,

(11) $$\Upsilon_{ij}=-\frac{K_{ij}}{V_{i}}-\frac{K_{ji}}{V_{j}}+\underset{l\notin \{i,j\}}{\sum }\frac{K_{li}K_{lj}}{V_{l}}.$$

Equation (11) can be used to compute the concentration coefficients corresponding to the TP and BF structural models. For instance, we have for the TP model

(12) $$\begin{array}{ll} \Upsilon_{12} & =-\frac{\lambda_{21}}{V_{2}},\\ \Upsilon_{13} & =0,\\ \Upsilon_{14} & =\frac{\lambda_{51}\lambda_{54}}{V_{5}}. \end{array}$$

From this example, we see that two cases can arise. In the first case (e.g., *Υ*_13_), the value of the concentration coefficient is equal to zero, not because of the specific numerical values that have been assigned to the path coefficients, but because of the structure of the SEM itself. In the second case (e.g., *Υ*_12_ or *Υ*_14_), the concentration coefficient is equal to zero only if the path coefficients are set to certain values (e.g, *λ*_21_ = 0 for *Υ*_12_; *λ*_51_ = 0 or *λ*_54_ = 0 for *Υ*_15_). For our purpose, the exact values taken by the nonzero *Υ*_*ij*_ are of minor importance; we rather focus on the elements that, such as *Υ*_13_, are structurally equal to zero, that is, that are equal to zero independently of the values taken by the path coefficients. More generally, it can be shown using (11) that *Υ*_*ij*_ is identically equal to zero regardless of the numerical values of the path coefficients if and only if the three terms of the right-hand side of (11) are equal to zero, that is,

(*C*_1_) *K*_*ij*_ = 0 and *K*_*ji*_ = 0: neither region *i* nor region *j* has an effect on each other;
(*C*_2_) *K*_*li*_ *K*_*lj*_ = 0: regions *i* and *j* do not jointly influence region *l*, for all *l* ≠ *i*, *j*.

**Figure 2:**
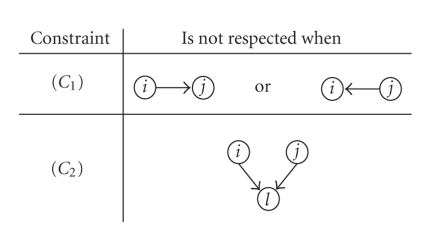
Structures that render either constraint (*C*_1_) or (*C*_2_) invalid for the pair *i*-*j*, thereby leading to *Υ*_*ij*_ ≠ 0 or, equivalently, Π_*ij*_ ≠ 0.

In other words, *Υ*_*ij*_ = 0 if and only if there are no such structures as *i* → *j*, *i* ← *j*, or *i* → *l* ← *j* for any l in the structural graph: according to (*C*_1_), there is no structural connection between *i* and *j* and, according to (*C*_2_), regions *i* and *j* do not jointly influence a third region *l*. When a pair of regions is not directly connected in the structural model or both regions do not jointly point to any common region, the coefficient of partial correlation between these two regions is expected to be structurally equal to zero. On the other hand, if either condition is not satisfied, the corresponding coefficient of partial correlation is not structurally equal to zero (see Figure 2). Turning our attention back to the TP model, we see that, while regions VEC and SMA satisfy both (*C*_1_) and (*C*_2_) (implying Π_13_ = 0), regions VEC and PFC do not satisfy (*C*_1_) (since we have VEC → PFC) and regions VEC and IFG do not satisfy (*C*_2_) (since we have VEC → IPL ← IFG). As a matter of fact, all cases can be found in both the TP and the BF models, as shown in Tables 2  and 3. Using the aforementioned rule, we are able to retrieve the following structural constraints for partial correlation:

- for the TP model: *Υ*_13_ = *Υ*_24_ = *Υ*_25_ = *Υ*_35_ = 0;
- for the BF model: *Υ*_13_ = *Υ*_14_ = *Υ*_25_ = 0.

### 2.5. SEM and Partial Correlation

As correlation matrices are often easier to interpret than covariance matrices, we can decide to examine partial correlation matrices rather than concentration matrices. The partial correlation coefficient between two regions *i* and *j*, denoted by Π_*ij*_, is here defined as a particular conditional correlation coefficient; it is the correlation between these two regions conditioned on the set of remaining regions, that is,

(13) $$\Pi_{ij}=\text{Corr}\left[y_{i},y_{j}\;|\;y_{\mathbb{R}\setminus \{i,j\}}\right].$$

There are hence *D*(*D* − 1)/2 partial correlation coefficients (10 in our example) that form the *D*-by-*D* partial correlation matrix **Π** = (Π_*ij*_). **Π** can readily be calculated from **Υ** through the following relationship [17]:

(14) $$\Pi_{ij}=-\frac{\Upsilon_{ij}}{\sqrt{\Upsilon_{ii}\cdot \Upsilon_{jj}}}$$

for two distinct regions *i* and *j*, and Π_*ii*_ = 1. Consequently, we have

(15) $$\Upsilon_{ij}=0⟺\Pi_{ij}=0,$$

and what has been said about the relationship between the structural model and the structural zeros of the concentration matrix, namely conditions (*C*_1_) and (*C*_2_), also holds for the partial correlation matrix. Furthermore, since the partial correlation coefficients are correlation coefficients, they are not influenced by any scale effect and remain between −1 and 1; for this reason, they are much easier to analyze and interpret than elements of the concentration matrix.

## 3. Validating Partial Correlation Structures

As we saw, a structural model has unique implications in terms of the structural pattern of partial correlation that can be expected from the data. Since the partial correlation matrix is a quantity that can be inferred from the data, we can use partial correlation analysis as a way to validate a structural model by comparing what is expected and what is observed.

### 3.1. Local Hypotheses

The approach consists of translating the structural hypotheses in terms of partial correlation. Indeed, according to Tables 2  and 3, the two structural models entail different hypotheses in term of partial correlation. For the theoretically preferred model, we have

(16) $$\begin{array}{l} \Pi_{13}=0\;\left(H_{\text{TP}1}\right),\\ \Pi_{24}=0\;\left(H_{\text{TP}2}\right),\\ \Pi_{25}=0\;\left(H_{\text{TP}3}\right),\\ \Pi_{35}=0\;\left(H_{\text{TP}4}\right), \end{array}$$

and, for the best fit model,

(17) $$\begin{array}{l} \Pi_{13}=0\;\left(H_{\text{BF}1}\right),\\ \Pi_{14}=0\;\left(H_{\text{BF}2}\right),\\ \Pi_{25}=0\;\left(H_{\text{BF}3}\right). \end{array}$$

While some hypotheses are identical for both models, (*H*_TP1_) = (*H*_BF1_) and (*H*_TP3_) = (*H*_BF3_), others have no equivalent in the other model, such as (*H*_TP1_), (*H*_TP4_), and (*H*_BF2_). The objective is then to infer the validity of these hypotheses with regard to the data.

### 3.2. Inference

Assessing the validity of the various hypotheses can be done by first estimating the partial correlation matrix. Inference of **Π** can be performed in a Bayesian framework using a numerical sampling scheme ([11, 13], see also the appendix). While direct computation of p(**Π** | **z**) is rather complex, this technique provides a simple approximation by sampling *L* (e.g., *L* = 5000) matrices (**Π** ^[*l*]^)_*l*=1,…,*L*_ from p(**Π** | *z*). We then quantify the relevance of all hypotheses as follows. First, the probability of a coefficient Π_*ij*_ to be higher than 0 can be approximated by

(18) $$p_{ij}^{+}=\text{Pr}\left(\Pi_{ij}>0\right)\approx \frac{1}{L}\#\left\{l:\Pi_{ij}^{\left[l\right]}>0\right\},$$

where “#” stands for the cardinal of a set (i.e., its size). The probability *p*_*ij*_^− ^ of a coefficient to be lower than 0 could be approximated in a similar way, but only one of these two quantities need to be computed, since we have *p*_*ij*_^+^ + *p*_*ij*_^−^ = 1. From there, the bearing of having Π_*ij*_ > 0 can be quantified by the log-odd ratio

(19) $$e_{ij}=10\;{\log\;}_{10}\frac{p_{ij}^{+}}{p_{ij}^{-}}=10\;{\log\;}_{10}\frac{p_{ij}^{+}}{1-p_{ij}^{+}}.$$

If *e*_*ij*_ is large and positive, we are more inclined to accept Π_*ij*_ > 0, while, if it is large and negative, we are more inclined to accept Π_*ij*_ < 0. Usually, a value of 10 dB can be considered as good evidence in favor of the hypothesis (see Table 4 for some relationships between *p*_*ij*_^+^ and *e*_*ij*_). We finally take |*e*_*ij*_| as a measure of how Π_*ij*_ differs from zero and, hence, as a way to quantify the deviation of the data from hypothesis Π_*ij*_ = 0: values close to zero indicate a coefficient close to zero, while large values suggest a large coefficient value. 

 Since we here focus on the partial correlation constraints entailed by the structural models, (16) and (17), we only need the corresponding log odd ratios, summarized in Table 5. If all these hypotheses were true, then we would expect the absolute values of all log odd ratios to be lower than 10 dB. While this is the case for the three hypotheses related to the BF model, it is not the case for two of the four hypotheses related to the TP model: according to these results, (*H*_TP2_) and (*H*_TP4_) are rather unlikely to be true. 

## 4. Discussion and Perspectives

In this paper, we further examined how partial correlation could be used to investigate effective connectivity in fMRI. We introduced theoretical fundaments explaining why and in what measure the structure of the partial correlation matrix can be related to a structural model. More precisely, we showed that, given a structural model, the partial correlation Π_*ij*_ between *i* and *j* is structurally equal to zero if and only if (*C*_1_) neither region *i* nor region *j* has an effect on each other, and (*C*_2_) regions *i* and *j* do not jointly influence a third region *l*; in other words, if and only if none of the following patterns are observed: *i* ← *j*, *i* → *j*, or *i* → *l* ← *j* for any *l*. From there, the definition of a structural model entails a unique set of constraints that can be tested from the data, supporting or invalidating the plausibility of the corresponding structural model. 

 When examining the global relevance of partial correlation analysis to the investigation of effective connectivity, we must jointly consider two complementary effects, namely, the theoretical relationship between structural models and partial correlation matrices on the one hand and, on the other hand, the quality of the inference process. From a purely theoretical standpoint, this result shows that partial correlation analysis comes up as a combination of two effects. First, constraints (*C*_1_) and (*C*_2_) imply that

(20) $$\Pi_{ij}=0\Rightarrow \neg \left(i\leftarrow j\right),\;\;\neg \left(i\to j\right),$$

where ¬ stands for the negation. In other words, a zero partial correlation between *i* and *j* implies the absence of a direct link between these two regions. Were there only (*C*_1_), this implication would be an equivalence and having Π_*ij*_ ≠ 0 would imply a direct link between *i* and *j*. However, this is not true in general and, more specifically, for any pair of regions for which constraint (*C*_2_) is satisfied. Such pairs are not connected but still have a nonzero partial correlation coefficient. As a consequence, all that can be said is that the set of set of pairs of regions with a zero partial correlations is a subset of the sets of pairs not directly connected in the structural model or, equivalently, that the set of pairs of regions connected in the structural model is a subset of the set of pairs of regions with a nonzero partial correlations. These features can easily be related to basic graph theoretic concepts. Condition (*C*_1_) states that regions *i* and *j* are not neighbors; condition (*C*_2_) states that *i* and *j* satisfy the so-called Wermuth condition [17]. As a consequence, the partial correlation constraints imposed by a structural model can be read off the graph obtained by adding undirected edges to eliminate all Wermuth configurations (for condition (*C*_2_)) and transforming all arrows into undirected edges (for condition (*C*_1_)). Such a graph is called the moral graph associated with the structural model. Depending on how many variables share common parents, the moral graph can be more or less close to the structural graph. For instance, in each of the two models used in this paper, condition (*C*_2_) was only met once. Whether this is a general feature of fMRI data or only a characteristic induced by the structure selected remains to be cleared. 

 Another theoretical issue that needs to be tackled is the fact that having a partial correlation that is not constrained to 0 (e.g., Π_14_ for the theoretically preferred model) does not preclude its value to be equal to zero, due to a numerical coincidence. Indeed, (11) shows that specific values of **K** and **V** could be selected to induce *Υ*_*ij*_ = 0 and, consequently, also Π_*ij*_ = 0. Even though this event is possible, it should be considered as rather unlikely, unless there is an underlying constraint at stake that forces the coefficient values to respect a certain relationship. 

 Another, more important issue deals with inference and how confident we can be in the partial correlation estimates and, critically, in the tests that their values are different from zero. The major difference between partial correlation and marginal correlation is that the former is obtained by removing the effect of *D* − 2 regions as evidenced by (14). Importantly, the partialization process tends to decrease the value of correlation regardless of the exact relationship between the two variables and the conditioning set. Consequently, the values of partial correlation coefficients usually tend to be lower than their marginal counterparts; this is an observation that we have made consistently, and with only few exceptions. Also, as a rule of thumb, the posterior variance associated with a (marginal or partial) correlation coefficient (e.g., Var[Π_*ij*_ | **y**] for partial correlation) is roughly a decreasing function of the absolute value of its posterior mean (e.g., E[Π_*ij*_ | **y**] for partial correlation). For instance, it is asymptotically (1 − Π_*ij*_^2^)^2^/(*N* − 1) (which is indeed a decreasing function of Π_*ij*_) for partial correlation and a similar result hold for marginal correlation [15]. A lower mean value therefore also implies a higher variance and, essentially, a bigger difficulty to discriminate a nonzero value from zero. 

 Altogether, these various factors, both theoretical and inferential, have different consequences on the relationship between the inferred pattern of partial correlation and the underlying structural model. Although we have observed a rather good agreement between expected and inferred patterns so far, in the lack of gold standard, these consequences must be further investigated. 

 Still, one of the main reasons why partial correlation analysis might become an important tool for the investigation of effective connectivity is that it is, to our knowledge, the only fully exploratory approach. Its key feature is its ability to retrieve local patterns of interaction. Indeed, while the method developed for the estimation of structural parameters, for example, (2), globally assesses the goodness of fit of the whole model and accordingly provides a general measure of it, partial correlation analysis provides a rather local assessment of effective connectivity, since the fact that two regions have a nonzero partial correlation depends on their connection with each other and of a potential connection with a common third region. For instance, in our example, while Bullmore et al. [7] concluded that the data did not contain enough evidence to prefer the BF model over the TP model (global statement), we showed that the TP model entails two partial correlation constraints (Π_24_ = 0 and Π_35_ = 0) that are rather unlikely to be true in the data (local statements). According to this result, we should discard the BF model or, at least, exert great caution when using it. Furthermore, if one only had the theoretically preferred model and were testing it, the large log odd ratios corresponding to hypotheses Π_24_ = 0 and Π_35_ = 0 would hint that the corresponding constraints might not hold and that there might be a direct connection between regions PFC and IFG on the one hand and, on the other hand, between regions SMA and IPL. 

 In this paper, we determined whether certain coefficients could be considered as different from zero or not in a Bayesian framework. This led us to the use of the evidence *e*_*ij*_ of (19). While increasingly used, evidence admittedly remains rather uncommon in the brain imaging literature, where significance is often asserted with respect to a significance threshold, or *P*-value. It would therefore be tempting to propose a direct connection between *P*-values and evidence or, at least, interpret results of our Bayesian approach in terms of significance and *P*-value (see, e.g., [12]). Unfortunately, doing so is both inaccurate and misleading, because of the strong difference between Bayesian probability intervals and their frequentist counterparts, confidence intervals. Under the null hypothesis (*H*_0_): Π_*ij*_ = 0, thresholding a statistic ${\hat{\Pi }}_{ij}$ at 10% in a frequentist framework (corresponding to a statistic of *P*_10%_) implies that, assuming that (*H*_0_) is true, there is only 10% to obtain data with a statistic above the threshold, that is,

(21) $$\text{p}\left({\hat{\Pi }}_{ij}>P_{10\text{\%}}\;|\;H_{0}\right)=0.10.$$

In this case, there is no mention whatsoever of any alternative hypothesis: we only assess how typical the data under consideration are. By contrast, thresholding a Bayesian probability at 10% means that we only consider cases where the alternative hypothesis (*H*_1_) of (*H*_0_): Π_*ij*_ ≤ 0 has a probability of more that 0.9, that is,

(22) $$\text{p}\left(H_{1}\;|\;P_{ij}\right)>0.90.$$

While a frequentist threshold of 10% might appear permissive, a Bayesian threshold of 10% is already conservative, since it implies that (*H*_1_) is about 10 times more probable than (*H*_0_). For more details on this topic, the reader can refer to Jaynes [18]. 

 A last question is the possibility to apply partial correlation to other imaging modalities, such as electroencephalography (EEG) and magnetoencephalography (MEG). While the issue of removing the effect of other regions when considering the interactions between two regions remains relevant, whether partial correlation as defined here can provide a cogent solution remains to be investigated. One of the major properties of the fMRI signal is that, due to the convolution with the hemodynamic response, the temporal information that it conveys is usually considered as less relevant than in EEG or MEG. This is one of the major reasons why most EEG or MEG analyses are performed in the frequency domain. Of interest would therefore be to use partial correlation in this frequency domain. This analysis could be performed over time windows that are narrow enough to assume stationarity of the signal. How such an approach could be related to partial coherence [19, 20] remains to be clarified.

## Figures and Tables

**Table 1:** Sample marginal correlation coefficients of the real data set examined in Bullmore et al. [7].

		(1)	(2)	(3)	(4)	(5)
		VEC	PFC	SMA	IFG	IPL
(1)	VEC	1				
(2)	PFC	0.661	1			
(3)	SMA	0.525	0.660	1		
(4)	IFG	0.486	0.507	0.437	1	
(5)	IPL	0.731	0.630	0.558	0.517	1

**Table 2:**
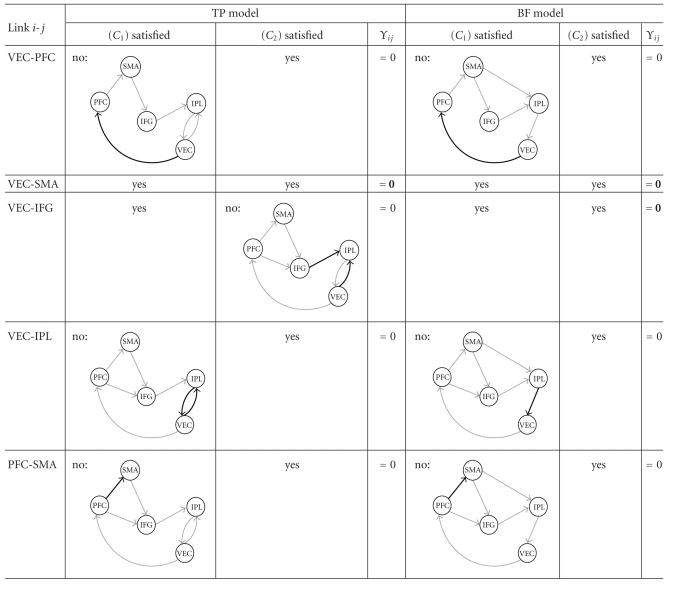
Partial correlation constraints in the TP and BF models (1/2). For each link between regions and each model, examination of whether (*C*_1_) and (*C*_2_) are satisfied.

**Table 3:**
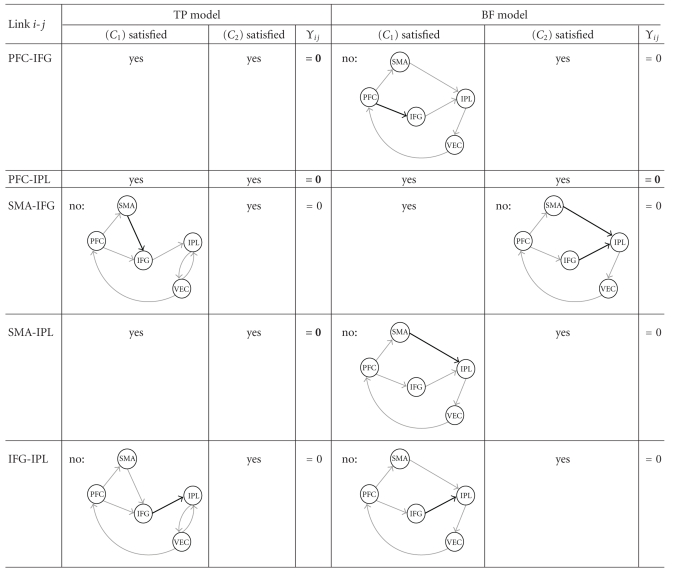
Partial correlation constraints in the TP and BF models (2/2). For each link between regions and each model, examination of whether (*C*_1_) and (*C*_2_) are satisfied.

**Table 4:** Evidence *e*_*ij*_ and probability *p*_*ij*_^+^ (from [16]). For evidences of 3 and 6, *p*_*ij*_^+^ is only approximately equal to the fraction.

*e*_*ij*_ (dB)	*p*_*ij*_^+^
0	1/2 = 0.50
3	2/3 ≈ 0.67
6	4/5 = 0.80
10	10/11 ≈ 0.91
20	100/101 ≈ 0.99
30	1000/1001 ≈ 0.999
40	10000/10001 ≈ 0.9999

**Table 5:** Real data. Relevance of hypotheses related to the TP and the BF models, respectively. Log odd ratios above a threshold of 10 dB are represented in bold.

Structural model	Constituting hypotheses	Structural constraints	|*e*_*ij*_|
TP	(*H*_TP1_)	Π_13_ = 0	1.6 dB
	(*H*_TP2_)	Π_24_ = 0	** 12.4 dB **
	(*H*_TP3_)	Π_25_ = 0	9.7 dB
	(*H*_TP4_)	Π_35_ = 0	** 13.1 dB**

BF	(*H*_BF1_)	Π_13_ = 0	1.6 dB
	(*H*_BF2_)	Π_14_ = 0	6.4 dB
	(*H*_BF3_)	Π_25_ = 0	9.7 dB

## Appendix

### Numerical Sampling Scheme

Using standard Bayesian theory, it can be shown that the covariance matrix **Σ** given the data *z* follows an inverse Wishart distribution with *T* − 1 degrees of freedom and scale matrix **U** = **S** ^−1^, where

(A.1) $$S=\overset{T}{\underset{t=1}{\sum }}\left(z_{t}-\bar{z_{t}}\right){\left(z_{t}-\bar{z_{t}}\right)}^{\text{t}}$$

is proportional to the sample covariance matrix, and $\bar{z_{t}}$ is the temporal mean [21]. Calculation of the posterior probability density function (pdf) of the partial correlation matrix, p(**Π** | **z**) cannot be performed in close form from this distribution. To approximate this distribution, we can nevertheless resort to the following sampling scheme [11, 13]. For sample *l*,

1. sample **Σ** ^[*l*]^ according to its inverse Wishart distribution ([21], Appendix A);
2. calculate **Υ** ^[*l*]^ = (**Σ** ^[*l*]^)^−1^, and **Π** ^[*l*]^ from **Υ** ^[*l*]^ according to (14).

Once a large number *L* of samples have been drawn following this process, the marginal pdf of a given quantity can be approximated by the frequency histogram obtained from the sample. Likewise, all statistics and estimators can be approximated by their sample counterparts. For instance,

(A.2) $$\begin{array}{c} \text{E}\left[\Pi_{ij}\;|\;z\right]\approx M_{ij}=\frac{1}{L}\overset{L}{\underset{l=1}{\sum }}\Pi_{ij}^{\left[l\right]},\\ \text{Var}\left[\Pi_{ij}\;|\;z\right]\approx X_{ij}=\frac{1}{L}\overset{L}{\underset{l=1}{\sum }}{\left(\Pi_{ij}^{\left[l\right]}-M_{ij}\right)}^{2}. \end{array}$$

One can also compute evidence as explained in the main text.
